# Selections

**Published:** 1897-11

**Authors:** 


### SELECTIONS.

An old and wornout horse, turned out to die, wandered along the fair banks of a babbling brook—he was so tired. He slowly walked, now near, now farther from the silvered music of the stream. The crimson and golden luxuriance of nature moved pathetically, and tear-drops of dew wet the ground, as a band of passing elves espied the faltering footsteps, and as the glories of a Western sky shone radiantly about the scene, sang this lullaby:

Blow, gentle breezes, blow,
Flow, peaceful waters, flow,
The burning heat of noon is past
And falls the evening shadows fast.
Blow, gentle breezes, blow.

Flow, peaceful waters, flow,
And bend ye willows low;
List to the song from off the shore
Above the distant waters roar.
Blow, gentle breezes, blow.

Flow, peaceful waters, flow,
As fall those footsteps slow;
Glide to the all-surrounding deep,
Rocked in its buoyant grandeur sleep.
Flow, peaceful waters, flow.

Blow, gentle breezes, blow,
Blow o'er the gathering woe;
Keen grow wounds on expectant years,
A crimson tide o'er banished tears.
Blow, gentle breezes, blow.

Flow, peaceful waters, flow,
Alike for friend or foe,
(What has been dimmed fancy's green,
Clear in a second-sight is seen.)
Flow, peaceful waters, flow.

Blow, gentle breezes, blow,
And o'er the dumb heart throw
A balm from off thy billowed crest,
Ere lay those weary limbs at rest.
Blow, gentle breezes, blow.

Blow, gentle breezes, blow,
Flow, peaceful waters, flow,
Humility can add but grace;
Thou earn, thou win, a finished race.
Blow, gentle breezes, blow.

F. B. BRUBAKER, M.D.

---

PROVOST C. C. HARRISON, of the University of Pennsylvania, in referring to the School of Veterinary Medicine in his opening address, October 1st, said:

"I shall ask the Trustees to take up the question of administering the Veterinary Department upon a greatly enlarged plan, suitable to the great importance of your branch of science. Agriculture is the foundation industry of the country. More than one-half of our population depends directly upon it, and the whole population indirectly. The prosperity of the farmer is the index and foundation of general prosperity. Agriculture depends in large measure on animal husbandry. Most of the highest priced and most concentrated products of the farm are animals or their products. The domestic animals of the United States represent investments of more than $2,500,000,000. Animals have their diseases as men have, and some plagues have swept them away by thousands, and have brought communities to the verge of bankruptcy. Many of their diseases are communicable to human beings, and are conveyed by contact or through the meat and milk. Hence the subject of veterinary science is of the utmost importance, both from the standpoint of public economy and of public health. It is the office of the Veterinary School to prepare men to take a leading part in the development of the productive resources of the country by improving and protecting domestic animals, and to fill important positions as guardians of the public health. It is estimated that the average efficiency of our farm-animals is not more than one-half of that of which it is capable under improved conditions, and as a result of more intelligent husbandry. This indicates the need of comparative pathological study and of practical zoötechnics which the Veterinary Department undertakes to give, and to do this thoroughly will require a more complete equipment than we now possess. I am sure that the importance of the subject, the zeal and industry of our Faculty and the prospect of sending perfectly equipped veterinarians in the world will justify the serious expense which will be involved in the execution of our plans.

"Meanwhile, let us here and all of us, each in his station, resolve that this shall be a faithful year to the utmost of our strength and talents, and may God speed us all in the several works which we this day begin."

At the conclusion of the Provost's address announcements were made by Dean Marshall for the Medical School, and by Dean Kirk for the School of Dentistry. Provost Harrison then introduced to the students Dr. Leonard Pearson, Professor of the Theory and Practice of Veterinary Medicine, as the new Dean of the Veterinary Department, stating that the additional duties imposed upon Dr. Marshall by his election to the Professorship of Chemistry made the change necessary. Dr. Pearson was received by the students with a round of applause. He made the announcements for his department.

---

THE WILD TAMARIND.—A curious plant is the wild tamarind, or jumbai plant (*Leucœna glauca*), of the river sides and waste places of tropical America; and very strange are its effects upon the non-ruminant animals that feed upon its young shoots, leaves, pods, and seeds, as described in the British Association by Mr. D. Morris, of Kew Gardens. It causes the horses to lose the hair from their manes and tails, has a similar effect upon mules and donkeys, and reduces pigs to complete nakedness. Horses are said to recover when fed exclusively on corn and grass, but the new hair is of different color and texture from the old, so that the animal is never quite the same as it was. One instance is cited in which the animal lost its hoofs too, and had to be kept in slings till they grew again and hardened. Ruminant animals are not thus affected, and the growth of the plant is actually encouraged in the Bahamas as a fodder plant for cattle, sheep, and goats. The difference in its action upon ruminants and non-ruminants is probably due to changes effected upon it in the chewing of the cud.—*Popular Science Monthly.*

---

An English farmer relieves choking by a stick four feet long and half an inch thick at the large end, with prongs, like fork-tines, about one inch long, at the small end. The stick should be short and smooth; wind the prongs with yarn until well covered, and sew over and through this a piece of cotton cloth, making a ball some inches in diameter, securely fastened to the small end of the stick; grease the ball well with lard, insert it in the animal's throat, and push it down the length of the stick if need be, or until the substance is forced into the stomach.

